# Modelling serial clustering and inter‐annual variability of European winter windstorms based on large‐scale drivers

**DOI:** 10.1002/joc.5481

**Published:** 2018-03-23

**Authors:** Michael A. Walz, Daniel J. Befort, Nicolas Otto Kirchner‐Bossi, Uwe Ulbrich, Gregor C. Leckebusch

**Affiliations:** ^1^ School of Geography, Earth and Environmental Science University of Birmingham Birmingham UK; ^2^ School of Civil Engineering and Geosciences Newcastle University Newcastle UK; ^3^ Institute for Meteorology Freie Universität Berlin Berlin Germany

**Keywords:** extratropical cyclones, large‐scale drivers, North Atlantic Oscillation, Poisson model, Scandinavian pattern, serial clustering, statistical modelling, windstorms

## Abstract

Winter windstorms are known to be among the most dangerous and loss intensive natural hazards in Europe. In order to gain a better understanding of their variability and driving mechanisms, this study analyses the temporal variability which is often referred to as serial or seasonal clustering. This is realized by developing a statistical model relating the winter storm counts to known teleconnection patterns affecting European weather and climate conditions (e.g., North Atlantic Oscillation [NAO], Scandinavian pattern [SCA], etc.). The statistical model is developed via a stepwise Poisson regression approach that is applied to windstorm counts and large‐scale indices retrieved from the ERA‐20C reanalysis. Significant large‐scale drivers accountable for the inter‐annual variability of storms for several European regions are identified and compared. In addition to the SCA and the NAO which are found to be the essential drivers for most areas within the European domain, other teleconnections (e.g., East Atlantic pattern) are found to be more significant for the inter‐annual variability in certain regions.

Furthermore, the statistical model allows an estimation of the expected number of storms per winter season and also whether a season has the characteristic of being what we define an active or inactive season. The statistical model reveals high skill particularly over British Isles and central Europe; however, even for regions with less frequent storm events (e.g., southern and eastern Europe) the model shows adequate positive skill. This feature could be of specific interest for the actuarial sector.

## 1 INTRODUCTION

Winter windstorms embody a prominent feature of the European climate. They are often accompanied by severe surface winds that can result in extensive socio‐economic losses. More than half of the insured loss caused by natural hazards in central Europe emanates from extreme winter windstorms (Munich Re, 2007). Various studies have discussed the potential forcing factors influencing the inter‐annual storm variability expressed in increased or decreased numbers of potentially destructive cyclone systems per season. An atmospheric variability pattern that is frequently considered in connection with the occurrence of winter storms is the North Atlantic Oscillation (NAO) (Hurrell, 1995; Walker, 1928). The association between the NAO and European storminess has been extensively examined in previous studies. Pinto *et al*. (2009) showed that a positive NAO phase is in favour of growth conditions of storms compared to a negative NAO phase. Likewise, Donat *et al*. (2010) found that more than 20% of storm days occur within a strongly positive NAO phase even though this period is found on less than 7% of all days. Other atmospheric teleconnections that have been detected as driving factors for the variability of the European winter climate include the East Atlantic/West Russia pattern (EA/WR) (e.g., Lim, 2015) or the Scandinavian pattern (SCA) (e.g., Bueh and Nakamura, 2007). Seierstad *et al*. (2007) related extratropical storminess, defined as monthly mean variance of high‐pass‐filtered sea level pressure, to large‐scale patterns by using a Gamma regression. They showed that five teleconnection patterns are significant at the 5% level with regard to explaining the inter‐annual variability: NAO, SCA, EA pattern, EA/WR and the Polar pattern (POL). More recently, Hunter *et al*. (2016) found a significant correlation between cyclone counts in Scandinavia and the SCA index. In terms of a physical link between large‐scale patterns and European storminess, Woollings and Blackburn (2002) could detect an influence of the NAO and the EA on the location and strength of the North Atlantic jet stream which is in turn responsible for increased or decreased storminess during the European winter. Other studies suggest that variations in the sea surface temperature (SST) in the North Atlantic also act as an important driver of variability of European storminess. Periods of high decadal storm activity were identified to be preceded by a phase of a weak North Atlantic meridional overturning circulation (MOC) by Nissen *et al*. (2014). This is due to a distinctive change in the mixed ocean layer heat content (OHC). Saunders and Qian (2002) and Czaja and Frankignoul (2002; 1999) found a Horseshoe‐shaped anomaly pattern of North Atlantic SST in summer and autumn that exhibited a strong link to the NAO in the subsequent winter. These findings could be supported by Renggli (2011), who showed that a horseshoe pattern in autumn is linked to windstorm frequency in the subsequent winter. One of the factors identified for the extreme storm season of the winter 2013/2014 by Wild *et al*. (2015) is the meridional temperature gradient between the North American continent and the SSTs in the West Atlantic. According to their study, there is a significant correlation between this temperature gradient and windstorm occurrences over the eastern Atlantic, the Iberian Peninsula and the southwest of the British Isles.

Understanding the serial clustering of winter windstorms is a key component in comprehending their inter‐annual variability. It is of particular interest within the actuarial industry as temporal clustering is responsible for large accumulated losses over an entire storm season. Seasonal clustering has been investigated statistically by Mailier *et al*. (2006), Vitolo *et al*. (2009) and Pinto *et al*. (2013). Their studies reveal the overall pattern of cyclone clustering (over‐dispersion) on both sides and downstream of the North Atlantic storm track, while under‐dispersion is found around the entrance of the storm track, hence close to Newfoundland. Recently, Pinto *et al*. (2016) showed that the statistical features of serial clustering and the influence of the NAO on serial clustering are independent from the storm/cyclone tracking algorithm used to identify the events.

The common statistical definition of seasonal clustering is to examine the deviation of a windstorm count time series from the Poisson distribution which features equal mean and variance. Thus, a natural approach is to verify to what extend the annual storm count time series follows a random point process or if the occurrences of storms are of a more systematic nature resulting in unequal mean and variance. This is realized by a dispersion statistic (variance to mean ratio) which is used to quantify the deviation from the random Poisson process. Vitolo *et al*. (2009), for example, found that monthly clustering is linked with the intensity of the storms. Thus, clustered seasons are likely to feature more intense windstorms than on average. Additionally, they were able to reproduce the dispersion statistic by using a Poisson regression model in connection with some of the large‐scale teleconnection patterns discussed above. Mailier *et al*. (2006) came to a similar result as they also identified five teleconnection patterns (NAO, EA, SCA, EA/WR and POL) that have a significant impact on the inter‐annual variability of cyclone counts for Europe, noting however that only the NAO by itself is not capable of explaining the entire variance. Economou *et al*. (2015) looked at the capability of 17 CMIP5 models to capture the clustering which is observed in the reanalysis data. In particular, in the northern and the southern part of the Atlantic storm track and over western Europe they were able to show the over‐dispersion of extreme windstorms counts. Additionally, they found that the variability of the NAO explains a large part of this over‐dispersion in the historical runs for the same areas. The question that arises from the results of these previous studies is, if serial clustering can be explained and modelled by a statistical model, thus if large‐scale drivers can be directly utilized to estimate the amount of windstorms per season and thus the serial clustering of the overall time series.

To examine these questions this article is aiming at answering two central questions:

1. Looking at different predefined regions in Europe: What are the main drivers responsible for serial clustering in a particular region?
2. After having identified several prominent drivers: Where is the main area of influence of these predominant European drivers on the inter‐annual variability of windstorms?

Question 1 is addressing the impact perspective of serial clustering as the regions are roughly in accordance with areas used within the actuarial sector whereas question 2 is examining this topic from a more physical angle: As the defined regions within the actuarial sector might split some of the areas of influence, our goal is to exactly locate the zone of influence of the dominant large‐scale drivers on the inter‐annual variability of windstorms. In order to precisely allocate drivers to different areas in Europe, we are answering question 2 on grid cell level, thereby creating a “map of drivers” for the European domain.

The intention of this study is to gain a better understanding and to quantify the inter‐annual variability of winter windstorm occurrence over Europe. This is achieved by investigating the influence of previously discussed teleconnections as well as testing further potential large‐scale drivers that have not been examined as thoroughly with regard to winter windstorms. Because of its importance for, for example, the insurance sector special focus is put on the link between these large‐scale drivers and serial clustering.

As the reanalysis product ECMWF ERA‐20C (Poli *et al.,* 2016) is relatively new, this investigation has never been carried out on the timescale of an entire century. We are aware of the potential handicap of ERA‐20C due to the lack of constraints for the reanalysis especially in the first half of the 20th century. As the scope of this study is solely the understanding of the physical drivers of windstorms, however, we leave the assessment and validation of this reanalysis product to further studies and also refer the reader to Befort *et al*. (2016) who compared the windstorm climatologies in ERA‐20C and NOAA 20CR (Compo *et al.,* 2011).

Additionally, all previous studies on serial clustering of winter storms (e.g., Mailier *et al.,* 2006; Vitolo *et al.,* 2009) utilized mean sea level pressure (MSLP) based tracking schemes to identify storm events whereas this study applies a wind‐based tracking algorithm. Additionally, the map of drivers could provide a useful overview over the spatial distribution of large‐scale drivers across the European domain.

## 2 DATA

**Figure 1:**
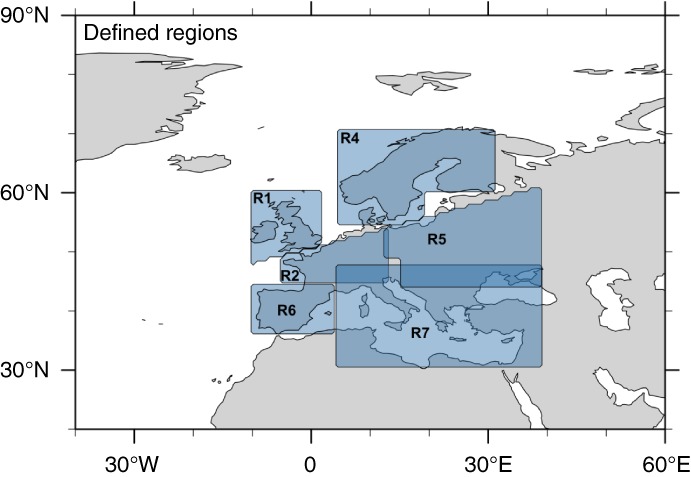
Defined European subregions. R1 = British Isles; R2 = central Europe; R3 = British Isles + central Europe (R1 + R2); R4 = Scandinavia; R5 = Eastern Europe; R6 = Iberia; R7 = Mediterranean

The ECMWF reanalysis ERA‐20C (Poli *et al.,* 2016) was used to identify windstorm events (featuring trajectories and footprints) for the core winter season December–February (DJF) during the past century using a wind tracking algorithm which is based on the local exceedance of the 98th percentile of wind speeds (Leckebusch *et al.,* 2008; Kruschke, 2015; Befort *et al.,* 2016). The identified windstorm trajectories are used to determine track densities of annual windstorm counts. This is performed either as simply counting annual windstorms passing through one of the seven regions (c.f., Figure 1) or, for the grid cell approach, as annual windstorm counts passing through a 500‐km radius around each 1° grid cell. Stalling or slow moving systems are only counted once for a respective grid cell ensuring a correct count number of storms. The respective time series of counts (either for the region or for the grid cell) is used as the predictant for the regression approach.

**Figure A1:**
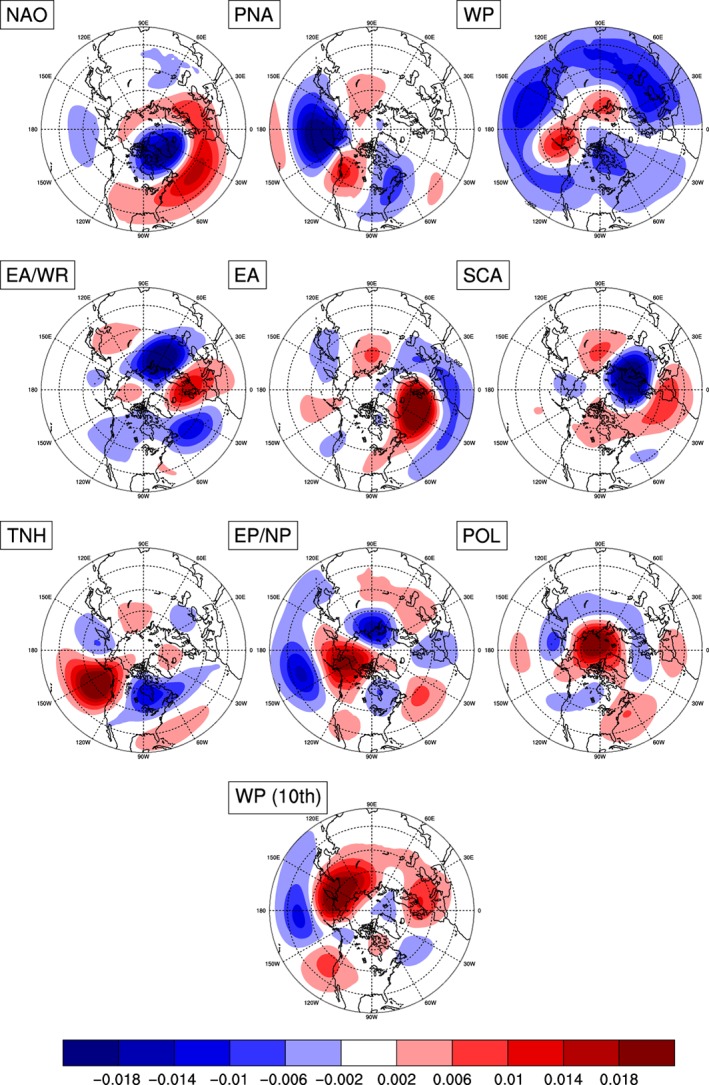
The 10 rotated EOF modes calculated from the 700‐hPa geopotential height field

The pool of potential large‐scale drivers (predictors) contains 20 normalized and standardized (by the standard deviation) index time series, including the 10 leading rotated empirical orthogonal functions (EOFs) (c.f., Figure A1 in Appendix) of 700 hPa geopotential height anomalies over the Northern Hemisphere (calculation according to http://www.cpc.ncep.noaa.gov/data/teledoc/telecontents.shtml), the station‐based NAO index and several SST‐related indices. Table 1 gives an overview of the nomenclature of the indices as used in the article. The complete list plus the definition used to derive each index can be found in Table A1 in Appendix. The quasi‐biennial oscillation (QBO) time series in the pool of potential drivers was kindly provided by Brönnimann *et al*. (2007) and is thus *not* calculated from the ERA‐20C reanalysis. The sea ice index time series is constructed by normalizing and standardizing the Northern Hemispheric sea ice extent between 40°N and 90°N during the core winter season (DJF) from ERA‐20C. The 20 predictors were tested for co‐linearity by calculating the variance inflation factor (VIF) (e.g., O’brien, 2007) for each potential predictor. The VIF reflects the proportion of variance in one predictor that is explained by all the other predictors in the model. In practice, the VIF is calculated for each variable as the reciprocal inverse of the coefficient of determination. A VIF of 1 would indicate no co‐linearity, whereas larger values suggest increasing co‐linearity between the predictors. The common threshold used as a cut‐off value is a VIF of 10 (e.g., Hair Jr. *et al.,* 1995).

**Table 1:** Large‐scale indices nomenclature as used in the article

Index name	Long name
QBO30	Quasi‐biennial oscillation (30 hPa)
QBO70	Quasi‐biennial oscillation (70 hPa)
AMO	Atlantic meridional oscillation
HSI	Horse shoe index
SSTS	Southern box of HSI
Tdif.Nam	Temperature difference North America–West Atlantic
W.Atl T	West Atlantic SST
NINO3.4	Nino 3.4 index
http://nao.is.li	Station‐based NAO index
PDO	Pacific decadal oscillation
West Pac	West Pacific pattern (EOF)
PNA	Pacific–North American pattern (EOF)
EOF10	West Pacific pattern II (EOF)
EA.WR	East Atlantic/west Russia pattern (EOF)
EA	East Atlantic pattern (EOF)
SCA	Scandinavian pattern (EOF)
TNH	Tropical Northern Hemisphere (EOF)
EP.NP	East Pacific/North Pacific pattern (EOF)
POL	Polar index
Sea ice	Northern Hemispheric sea ice cover

## 3 DEVELOPMENT OF THE STATISTICAL POISSON MODEL

This section shall provide an overview of the two perspectives used in this study: First, the regional, more impact‐based statistical model is introduced. Ensuing, the grid‐cell‐based Poisson model approach is described in detail.

In order to investigate regional characteristics of windstorm clustering in Europe, a statistical model is developed for seven different European subregions. These regions are defined by both meteorological and socio‐economic criteria which are widely used in the actuarial sector (c.f., Figure 1). As described in section 2, the amount of storms per core winter season (DJF) within the limits of a region is counted and used as the predictant in the stepwise regression approach.

The procedure of finding the main large‐scale drivers for every region is based on a stepwise AIC (an information theoretic criterion) approach (Akaike, 1974). The AIC is an information‐theory‐based (e.g., Jaynes, 1957) estimate of how much information is lost if using a statistical model instead of the actual physical relation. Thus, the AIC can be used as a tool for model selection when different models are available. Part of the selection process is the trade‐off between the goodness of fit of the model and the complexity of the model, as the number of parameters to be estimated *k*, as well as the maximum of the likelihood function *L* for estimating the regression coefficients are part of the AIC score (c.f., Equation (1)).

(1) AIC=2k−2lnL.

The model yielding the smallest AIC is declared as the chosen/winning model. Unlike other model selection criteria, for example, the *F* test, the AIC score does not provide any evidence about the absolute quality of the model. Thus, in case all available models are poor, there is still a winning model albeit it being of poor quality. To account for that the chosen predictors of the best AIC model are tested for statistical significance using a Wald or *χ* ^2^ test (e.g., Agresti, 2007). Only predictors significant at the 5% level are included in the final model.

The selected predictors for every region are further used for to fit a Poisson generalized linear model (GLM). As the Poisson GLM is the recognized model for modelling count data (e.g., Vitolo *et al.,* 2009) we consider it to be the natural choice. Because of a significant trend in windstorm counts identified in Befort *et al*. (2016), a linear time trend coefficient is added to the Poisson GLM. The model definition is given by Equation (2b):

*y*(*t*) represents the number of storm counts in season *t*, *λ*(*t*) represents the Poisson mean, *x*_1_(*t*) represents the time (108 DJF seasons) and the *x*_*i*_(*t*) represent the previously selected large‐scale drivers. The coefficients for the respective predictors are labelled with *β*_*i*_.

(2a) $$y\left(t\right)∼\text{Poisson}\left(\lambda \left(t\right)\right),$$

(2b) $$\log\left(\lambda \left(t\right)\right)=\beta_{0}+\beta_{1}x_{1}\left(t\right)+\sum_{i=2}^{N}\beta_{i}x_{i}\left(t\right).$$

The model is intended to capture the observed serial clustering given by the overall dispersion statistic over these seven regions. The clustering dispersion score (Mailier *et al.,* 2006) estimates how well the model is capable of reproducing the clustering of the entire time series. It is assessed through the index of dispersion (*D*), also referred to as the variance‐to‐mean ratio.

(3) $$D=\frac{\sigma^{2}}{\mu }-1.$$

The mean and variance are equal for an ideally Poisson distributed variable (leading to *D* = 0), thus the occurrence of an event is independent (in a statistical sense) of the timing of the previous event. This implies that a deviation of *D* from 0 suggests some kind of a serial dependence of successive statistical events. In terms of windstorms this indicates that successive storm seasons might not be independent of each other. This deviation of *D* from zero is the definition/quantification of irregularity, or in terms of windstorms, clustering. If the index of dispersion *D* is larger than 0 the time series is considered to be over‐dispersed (thus following a negative binomial distribution). Referring to the annual storm counts this implies that the occurrence of storms happens in clusters for individual years. The model for all seven different regions is compared via the clustering dispersion bias (*D*_model_ − *D*_obs_) thus, the difference between the modelled dispersion statistic and the actually observed dispersion statistic *D*, which is calculated from ERA‐20C directly.

As the dispersion statistic *D* by itself conveys no information about the clustering that happens within a particular season, we have decided to assess the regularity/irregularity of a particular season by a metric which we name *active* (AS) or *inactive* (IAS) season. A season is considered an AS if it spawns more windstorms than one standard deviation above the long‐term mean. Equivalently, a season is considered an IAS if it features fewer windstorms than one standard deviation below the mean.

(4) $${\mathrm{AS}}_{r,t}={\mathrm{Cts}}_{r,t}\vee {\mathrm{Cts}}_{r,t}>\mu_{r}+\sigma_{r},$$

(5) $${\mathrm{IAS}}_{r,t}={\mathrm{Cts}}_{r,t}\vee {\mathrm{Cts}}_{r,t}<\mu_{r}-\sigma_{r}.$$

Here *μ*_*r*_ represents the mean of the long‐term storm count time series in region *r* and *σ*_*r*_ represents its standard deviation. *Ctsr*_*r,t*_ represents the windstorm count for DJF *t* in region *r.* The amount of IAS/AS is compared for the observations and the model. This second metric represents an aspect of serial clustering that is more tailored towards the actuarial community as it conveys information on whether or not a season features above or below average storm counts, thus an increased or decreased likelihood of two or more storms in succession.

To examine in how far the Poisson GLM is able to reproduce these AS and IAS, we define a hit rate (HR): The HR is defined as the quotient of the count of correctly predicted and observed AS or IAS over the number of observed AS/IAS within the entire time series. This metric is used to assess the model’s ability to capture AS and IAS. The HR will be given as a percentage of correctly predicted AS. Thus, regardless of the actual count of storms, a season is considered to be predicted correctly if it exceeds the AS or IAS thresholds.

All comparisons are made to the observed windstorm counts derived from ERA‐20C. In order to account for potential over fitting a 10*‐*fold cross‐validation method (Krstajic *et al.,* 2014) is applied to the statistical model for all seven regions. The skill scores presented in the tables in section 4 are calculated using the cross‐validated model.

In order to examine the physical perspective of the influence of large‐scale drivers on the inter‐annual variability of windstorms (represented by track density) on grid cell level, we implemented an independent Poisson GLM approach for every grid cell using five predominant drivers which are partly taken from literature (e.g., Mailier *et al.,* 2006; Vitolo *et al.,* 2009) and partly from results of the impact‐based statistical model. As the intention is to comprehend annual windstorm count on grid box level, a Poisson regression model appears as the natural choice again (c.f., Equation (2b)). An annual track density of windstorms per core winter season (DJF) is calculated on a 1° grid cell level for the North Atlantic domain (40°W–40°E, 30°–80°N) for the 108 years of ERA‐20C data. Subsequently, a Poisson regression is carried out in which the track density time series per grid cell is regressed against the five defined large scales. The intention for performing so is to create a “map of drivers,” thus a spatial distribution of the predominant large‐scale drivers accountable for windstorms in the North Atlantic domain. The predominant drivers are identified by determining the significant predictor (using a *χ* ^2^ test at a 5% significance level) with the largest absolute regression coefficient (out of the five available drivers) of the Poisson for every grid cell, respectively. Jointly the five most common drivers of the serial clustering model appear as the predominant driver in over 95% of all grid cells. Results for the grid box level analysis will be presented in section 4.2.

## 4 RESULTS

### 4.1 Identified large‐scale drivers for serial clustering for seven European regions

The large‐scale drivers for the seven European regions that are identified by the Poisson GLM AIC approach are presented in Table 2. As we are applying a Poisson GLM model with a logarithmic link, the magnitude of the regression coefficient quantifies the relative importance of every selected driver. Thus, if, for example, regression coefficient *β*_2_ for the SCA pattern increased by 1 unit, the impact on the statistical model would be exp(*β*_2_ + 1) times higher. As a result this implies that the larger the regression coefficient, the higher the relative importance of the associated large‐scale driver. The regression coefficient *β*_1_ associated with the linear time trend is significantly positive across all seven regions, thereby confirming the identified trend in windstorm in Befort *et al*. (2016) for the entire domain.

**Table 2:** Selected drivers for the Poisson GLM modelling the serial clustering of windstorms. The drivers are sorted by the magnitude of their regression coefficient. Values above the dashed line represent positive values; values below the line are negative. The observed and modelled dispersion score *D* is given at the bottom of the table

	BIRegion 1	C.EurRegion 2	BI/C.EurRegion 3	ScandRegion 4	East E.Region 5	IPRegion 6	MedRegion 7
Selected large‐scale drivers	NAO stat SCA Tdif.Nam	SCA Tdif.Nam QBO30	SCA NAO stat Tdif.Nam EOF 10	SCA W.Atl.T West Pac	SCA EOF10 W.Atl.T EA.WR	EP.NP Tdif.Nam SCA	SCA EA Sea ice
	EA POL	EA EA.WR	/	PNA POL	PNA	POL NAO stat EA.WR EA	West Pac
Dispersion score *D* model	1.14	0.12	0.60	1.54	0.63	0.09	−0.30
Dispersion score *D* observed	1.94	0.85	1.35	2.67	1.53	0.97	0.51

Across all regions the SCA, the EA and the station‐based NAO index appear as the main drivers of serial clustering of windstorms as well as for the drivers of AS and IAS. The temperature gradient between the North American continent and the western Atlantic SSTs (introduced by Wild *et al.,* 2015) also proves to be of importance related to windstorm clustering especially for the British Isles, central Europe and the Iberian Peninsula. This is in accordance with their findings of a statistically significant correlation between this temperature gradient and windstorm counts over the Iberian Peninsula (Wild *et al.,* 2015). The selection of the QBO in 30 hPa for central Europe suggests a troposphere–stratosphere coupling that has an impact on the windstorm frequency for certain parts of Europe. Potentially, there is a link between the Northern Hemisphere polar vortex and the Arctic Oscillation which is in turn coupled to the NAO (Baldwin and Dunkerton, 2001).

Across all regions, except for the Iberian Peninsula and the British Isles, the SCA index appears as the main driver for windstorm variability. This is somewhat intriguing as previous studies (e.g., Pinto *et al.,* 2009; Donat *et al.,* 2010) identified the NAO as the prominent driver for European storminess. The NAO is identified as the leading driver for the British Isles which could lead to the conclusion that the influence of the NAO variability pattern is not as far stretched across Europe as the SCA pattern. The anti‐correlated impact of the NAO on serial clustering over the Iberian Peninsula supports the assumption of the NAO only having a significant impact for the far western parts of Europe. To further investigate the spatial distribution of some of the large‐scale drivers, we refer the reader to section 4.2 where we present the result for the grid‐cell‐based analysis for some selected large‐scale drivers (“map of drivers”).

In fact, most of the selected drivers originate from the EOF analysis of MSLP data in the Northern Hemisphere. An intriguing result is the selection of the Northern Hemispheric sea ice cover which is linked with clustering in the Mediterranean region which will be discussed in section 4.2. Interestingly, there is no negatively correlated driver identified for the joint region of the British Isles and central Europe although there is for the individual regions. This could be due to the increased size of the region and the mixing of drivers that would only influence the western or the eastern parts of that region. Situations like these motivate the map of drivers presented in section 4.2. Although focussing on Europe and the North Atlantic there are some variability modes, which have their centre of action more towards the Pacific region (i.e., PNA, West Pacific mode and EOF10). This emphasizes the importance of global teleconnections for winter windstorms, implying that in order to thoroughly comprehend the European winter climate analysis solely based on Europe might not suffice.

The dispersion index *D* is positive for all seven regions as given in the bottom two rows of Table 2. The largest observed values for *D* are found for Scandinavia, the British Isles and eastern Europe implying that the time series of windstorm counts in these regions are following a negative binomial distribution, thus featuring larger variance than mean. The modelled values for *D* are too small for all regions which connote an underestimation of the variance by the statistical model. The index of dispersion is close to zero for the Iberian Peninsula and negative for the Mediterranean region denoting that the variance there is smaller than the long‐term mean or, in statistical terms, the process is more regular than random.

**Figure 2:**
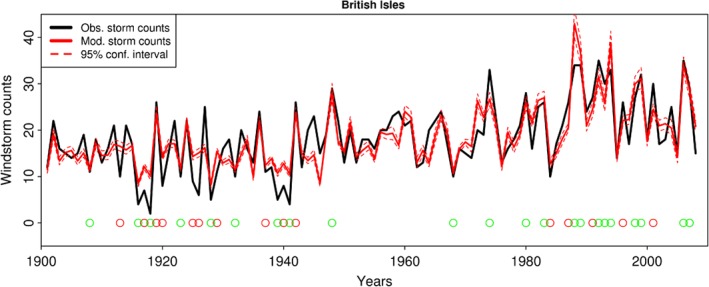
Windstorm frequency per year for the British Isles from 1901 to 2008. The observed counts are depicted in black; the Poisson GLM modelled counts in red. The red dashed line represents the 95% confidence interval. Circles indicate AS/IAS. Green circles mark seasons in which the AS/IAS are predicted correctly whereas red circles indicate years where the activeness of the season was not predicted correctly

The qualitative performance of the model can be assessed in Figure 2. It depicts the modelled as well as the observed windstorm counts per winter season for the British Isles region (region 1). The observed time series (black) is within the 95% confidence intervals for most of the years implying a good fit of the model. The large amount of green circles further implies the capability of the model to skilfully identify AS and IAS. The quantitative performance of the cross‐validated statistical model for the seven regions is displayed in Table 3. The average correlation between modelled and observed time series over all seven regions of 75% is remarkably good considering the application of a relatively simple Poisson GLM. The model works particularly well for the British Isles and the Scandinavian region where the correlation between modelled (cross‐validated) and observed time series is larger than 80%. Furthermore, the model explains up to 70% of the variability of the inter‐annual storminess. The British Isles and Scandinavia are also among the regions featuring the highest AS and IAS HRs. A stellar example is the 79% AS HR for the British Isles. The best IAS HR is found for the Mediterranean area. As discussed before, the modelled dispersion is generally smaller than the observed value throughout.

**Table 3:** Results for the cross‐validated metrics of the Poisson model performance. The highest value in each column is marked bold. ED, an equivalent to the *R* ^2^ value for linear regression models

Region	HR AS	HR IAS	Disp. score *D* bias model–obs	Correlation/ED
British Isles	**78.9%**	61.1%	−0.80	**0.84/0.70**
Central Europe	71.1%	50.0%	**−0.73**	0.72/0.51
BI+C.Europe	71.1**%**	65.5%	−0.75	0.81/0.64
Scandinavia	75.0%	64.3%	−1.13	0.82/0.67
Eastern Europe	68.8%	43.8%	−0.89	0.76/0.57
Iberian Pen	56.3%	41.2%	−0.88	0.66/0.44
Mediterranean	22.2%	**77.6%**	−0.81	0.63/0.40
Mean	63.3%	57.6%	−0.86	0.75/0.56

### 4.2 Map of drivers

**Figure 3:**
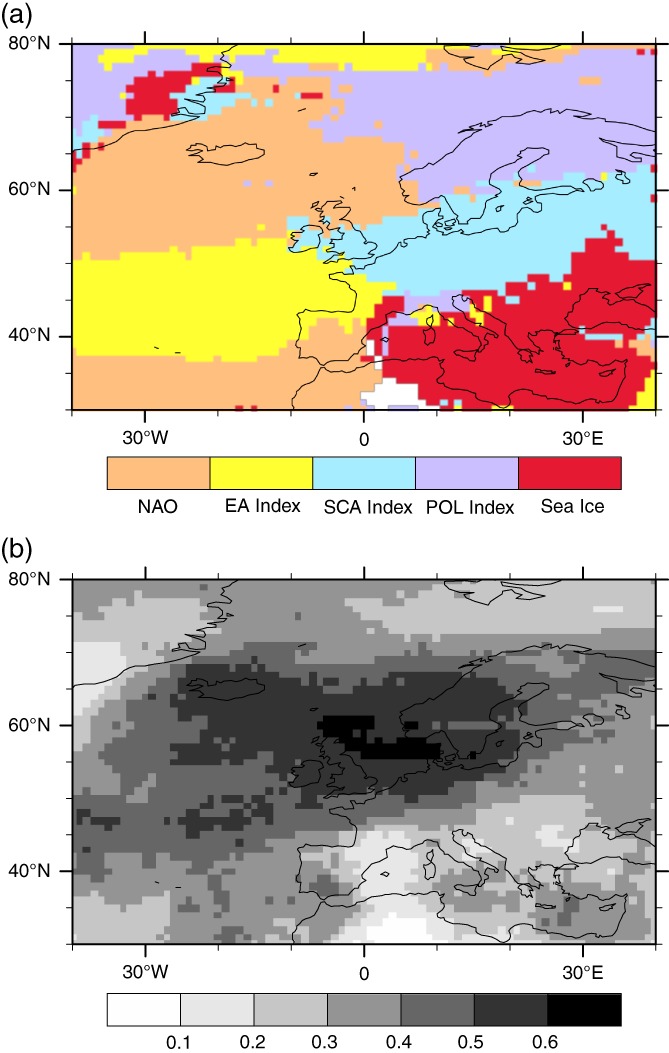
(a) Most dominant teleconnection patterns explaining the inter‐annual variability of windstorm counts per grid cell from 1901 to 2008 identified by a Poisson GLM. (b) Explained deviance of the Poisson regression model for every grid cell

Taking literature and our results from section 4.1 into account, we chose the NAO, the SCA, the EA, the POL and the sea ice time series as the overall leading variability patterns associated with winter windstorms for the European, the North Atlantic and the Mediterranean domain. The “map of drivers” created by grid‐cell‐based Poisson GLMs is presented in Figure 3a. The most important drivers for the variability of winter windstorms over main parts of the European mainland are the SCA and the NAO time series. The British Isles appear to be under the influence of several predominant predictors. While the southern part of the British Isles is influenced mostly by the SCA pattern, the northern part is more affected by the NAO. This is in line with the findings of section 4.1 where we showed that the leading driver for serial clustering over the British Isles is the station‐based NAO index time series. Because of co‐linearity naturally only one of the two NAO time series can be present in the pool of potential drivers. Although the EOF‐based NAO exhibits a correlation of over .8 with the station‐based index, the station‐based NAO index yielded the better results with regard to the statistical model. Figure 3, right depicts the explained deviance (ED) (McCullagh and Nelder, 1989) function of the Poisson distribution which represents an equivalent to the *R* ^2^ value of a linear regression, for example, it estimates how much variance is explained by the Poisson model in every grid cell. It is particularly high over the Northern Sea, Scandinavia and the East Atlantic with up to 60% variability jointly explained by the five large‐scale drivers. Generally, more windstorm variance is explained over the northern parts of Europe compared to the southern parts.

The extent of the Northern Hemispheric sea ice cover operates as a significant driver of storminess particularly for the central to eastern Mediterranean area and parts of the Black Sea. This is in line with the findings of section 4.1 where sea ice appeared as the second most important driver in this region, thereby also justifying the selection of sea ice as one of the main drivers for this grid‐cell‐based model.

An explanation for the sea ice as a significant driver could be the enhanced (weakened) meridional temperature gradient with more (less) sea ice extent as it is one of the factors that controls the stability of the mid‐latitude flow. Semmler *et al*. (2016) found a decreased number of wintertime cyclones in their sensitivity analysis with an 80% reduced Arctic sea ice extent over the eastern Mediterranean and parts of the Black Sea and also a reduced Eady growth rate over the Balkan region and the Black Sea (their Figures 12f and 14f, respectively). Our results indicate that larger sea ice extent leads to more windstorms, which is generally in line with those findings from Semmler *et al*. (2016). However, the interpretation of sea ice as a factor steering windstorm activity over this region should be performed with caution as only about 10–20% of the windstorm variability is explained by sea ice variability (Figure 3, right). It is also worth mentioning that there are several studies showing an opposite relationship between sea ice and cyclone/storm activity over the Mediterranean region (Grassi *et al.,* 2013). This indicates that further targeted studies are necessary to fully understand the link between Northern Hemisphere sea ice extend and storm activity over southern Europe, which is however beyond the scope of this study.

**Figure 4:**
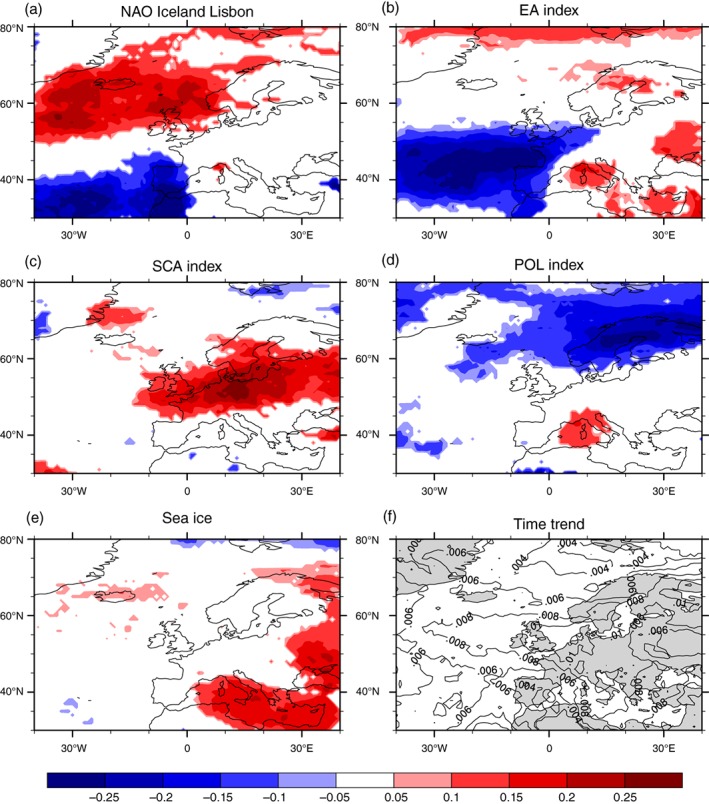
Significant Poisson GLM regression coefficients for the dominant selected large‐scale drivers explaining most of the variability of winter windstorms for Europe/North Atlantic (a)–(e) time trend coefficients of the Poisson GLM as contours (f). The contour levels for the time trend are separated by .002 intervals

As its name implies, the POL index serves as the main influence on inter‐annual windstorm counts in northern Scandinavia and the Polar regions. The EA index dominates the region between the two NAO poles which makes it the dominant driver for the eastern Atlantic as well as parts of western Europe, explaining the major part of the 60% inter‐annual variability for that area. The boundary between the NAO and the EA index, especially in the south, is remarkably well defined, implying that the area of influence of either of the predictors can be localized very precisely. It has to be noted that Figure 2 (left) only shows the “winning” large‐scale index yielding the largest absolute regression coefficient, for every grid cell. Most of the grid cells in the North Atlantic domain, however, feature more than one significant predictor. As the absolute value of the coefficients is depicted in Figure 3, it also has to be considered that some of the teleconnections (regression coefficients) are positive (northern pole of the NAO, SCA) and some are negative (southern pole of the NAO, EA index; c.f., Figure 4).

The panels in Figure 4 depict the regression coefficients of the five chosen teleconnection patterns and for the linear time trend as calculated by the Poisson GLM. The NAO exhibits the prominent dipole structure associated with the Icelandic Low and the Azores High. Its area of significant influence stretches across most parts of the Atlantic, the Iberian Peninsula, the British Isles and some parts of western Scandinavia. Strikingly, the European mainland is almost solely under the influence of the SCA teleconnection pattern (Figure 4c). This can be better understood by comparing the patterns of the two variability modes (Figure A1 in Appendix). The SCA and the NAO patterns look somewhat similar consisting of a negative pole in the north and a positive pole in the south. The SCA index, however, is shifted downstream so that its main centres of action are over Scandinavia and the Iberian Peninsula. This shifted dipole enables storm tracks to travel more directly eastwards and stretch further into the European mainland whereas the NAO leads storms on a more north easterly trajectory across Scandinavia. The regression coefficients of the POL index also appear as a weak dipole with its centres of action over the Mediterranean Sea and Scandinavia up to the Polar regions. The EA index is the dominant negative teleconnection pattern between the two dipoles of the NAO. Thus, during its negative phase the likelihood of observing more windstorms in the East Atlantic and western Europe is significantly increased. The area of significant coefficients coincides well with the positive centre of the EA pattern (c.f., Figure A1 in Appendix). The time trend is positive across almost the entire domain which implies a positive trend in windstorm counts. The strongest trend is found over the British Isles and Scandinavia whereas the trend over the Mediterranean area is considerably smaller.

In order to check for non‐stationary teleconnections, especially with regard to the Pacific (e.g., Greatbatch *et al.,* 2004), the drivers were also examined for two halves of the century separately (1901–1950 and 1951–2008; not shown). The results look very similar to the map presented in Figure 3a so that we can assume stationary teleconnection processes.

## 5 SUMMARY AND DISCUSSION

This study identifies and quantifies the impact of various large‐scale drivers on European winter windstorm counts and their seasonal clustering. For this purpose windstorm events were identified using an objective wind tracking algorithm (Kruschke, 2015; Befort *et al.,* 2016) in the 20th century ECMWF reanalysis ERA‐20C (Poli *et al.,* 2016). Befort *et al*. (2016) have shown that the high‐frequency variability of windstorms in ERA‐20C and NOAA 20CR (Compo *et al.,* 2011) is well correlated so that we can assume that the windstorm climatology in ERA‐20C and NOAA 20CR are in sufficient agreement for the scope of our study. We are fully aware of the potential issues regarding the century‐long reanalysis efforts especially for the beginning of the century. However, with this study we are not trying to validate and assess the quality of the reanalysis but solely trying to understand the drivers for the inter‐annual variability of windstorms.

The windstorm trajectories were used to count the annual amount of windstorms for seven defined European regions representing (a) diverse windstorm climatologies and (b) regions of interest within the actuarial community. The statistical model developed in section 3 is aimed at modelling serial clustering of windstorms over seven different European regions. The model is capable of assessing the inter‐annual variability in two different perspectives:

The clustering dispersion: Thus whether or not the variance‐to‐mean ratio is larger than 1 (according to Mailier *et al.,* 2006). In this case the time series is over dispersed which in return corresponds to the fact that occurrences (in this case storm counts) occur in clusters. The benefit of this approach is the understanding of which physical drivers/large‐scale modes are directly responsible for the deviation from the Poisson distribution, thus the storm counts in one season is more likely to be dependent on a previous season.

The modelling of the AS/IAS: Whether or not a single season spawns more or less than one standard deviation of storms compared to the long term mean. Ultimately, a skilful model w.r.t. modelling AS/IAS could be particularly beneficial for the insurance sector as windstorms over Europe are associated with extensive losses, especially if they occur in quick succession which is more likely in seasons with high storm counts.

The model is developed using a stepwise Poisson AIC approach to select the dominant drivers out of a pool of 20 large‐scale drivers that entail MSLP and SST related time series (c.f., Table 1). Compared to previous clustering studies (e.g., Mailier *et al.,* 2006); the investigated time series contained the entire 20th century, so that the results comprise a longer timescale than before. The regression coefficient associated with a linear time trend is significantly positive across all seven regions, hereby confirming the results of Befort *et al*. (2016).

Generally, the statistical model shows very satisfactory results regarding the goodness of fit of the windstorm count, the estimation of the dispersion score of the time series, and the ability to successfully define a respective season as AS or IAS across all seven regions. Especially the HRs of predicting AS for most of the regions are very promising. The results of this study suggest that a determinant proportion of the information, needed to accurately describe windstorm frequency and clustering, is being efficiently extracted from the set of large‐scale indices discussed, especially placing value on those indices selected for the different regions. The calculated scores confirm that the model is able to perform well even in regions with strongly diverse windstorm climatologies, for example, in the Mediterranean area or over the Iberian Peninsula. Although a lower overall performance is observed for those regions, most of the metrics assessed still show a satisfactory result (correlation >65% for the Iberian Peninsula), and more skill than a random forecast of HRs (>50% for the Mediterranean).

The five predominant drivers identified in literature and with our regional analysis were investigated in more detail on a grid box level via a Poisson GLM approach in order to produce a “map of drivers.” This map of drivers represents a spatial distribution of the predominant large‐scale driver for every grid cell across Europe and the North Atlantic. Thus, it can be used to deduct the influence of a respective driver for winter windstorm variability. These five predominant drivers entail NAO, SCA, POL, NH sea ice cover and the EA time series. These five teleconnection patterns represent significant drivers for more than 95% of all grid cells and jointly explain up to 60% of the inter‐annual windstorm variability (ED) over large parts of northern Europe and the Northern Sea.

This result is in good accordance with Seierstad *et al*. (2007) who also identify four out of these five large‐scale teleconnections with regard to European winter storminess. Instead of using actual tracked windstorm events, however, their storminess is defined as high‐pass‐filtered MSLP variance. Hence, we can confirm their findings based on a wind speed‐based definition of storminess. Their study reveals that the EA/WR pattern also has a significant impact on European storminess. Even though the EA/WR pattern is not found to be having an essential impact on grid cell level, it is identified as being significant for the statistical model for two out of the seven European regions which is in turn in overall accordance to their findings.

The identified drivers both for the impact and the physical‐based analysis are in good agreement with previous windstorm and windstorm clustering literature, confirming the SCA, the NAO, the EA and the POL time series as major drivers of the variability (c.f., Mailier *et al.,* 2006; Seierstad *et al.,* 2007; Pinto *et al.,* 2009; Vitolo *et al.,* 2009; Donat *et al.,* 2010). A striking result is represented by the importance of the SCA pattern both in the map of drivers and the impact analysis as it is identified as the most important driver for central Europe. This implies that when assessing the winter windstorm hazard in (central) Europe it seems as if it is not sufficient to focus on the NAO alone. The EA pattern is, as its name implies, the dominant feature for the East Atlantic including the northern part of the Iberian Peninsula and the southern part of the British Isles. The teleconnections can assumed to be stationary as the results for the split century analysis (1901–1950 and 1951–2008) show very similar results to the map of drivers comprising the entire century from 1901 to 2008.

Additionally to the previously studied drivers, we were able to identify the temperature gradient between the North American continent and the West Atlantic (Wild *et al.,* 2015) and the Northern Hemispheric sea ice extent as locally important drivers (Iberian Peninsula and Mediterranean). Sea ice cover as a teleconnection with the Mediterranean is generally in line with results from Semmler *et al*. (2016) who found a reduced number of cyclones in the eastern part of the Mediterranean and over the Black Sea in their sensitivity study with reduced sea ice. We argue that we observe the same direction of that correlation, thus more sea ice could lead to an increased Eady growth rate and higher numbers of windstorms in the eastern Mediterranean and the Black Sea area. We assume that the storminess in this area is linked with an enhanced meridional temperature gradient over the North Atlantic. Sea ice however still has to be treated with caution as we could show that it only makes up for about 10–20% of the ED in the respective region. The maximum of this ED can be found in areas where there is also some influence from the EA and the POL which both show weak dipole structures over the Mediterranean (c.f., Figures 3b and 4b,d).

It should be noted that neither the model developed on grid box level nor the model for the seven regions include interactions between the predictors. An inclusion of those might improve the fit/skill of the regression/model; however, it proves to be more demanding to identify the impact of individual predictors. For that reason, the interactions between the predictors have not been considered. However, regarding future work this is definitely an approach worth investigating in more detail, especially for the development of a more sophisticated statistical model. Considering the time trend of the Poisson model, a quadratic or even exponential trend could be tested and compared to the linear trend.

Albeit the model being of relatively simple nature, it produces more than satisfactory results, particularly with regard to the identification of large‐scale drivers for the seven defined regions and the HRs for most of the seven regions (correlations >80%, HR > 70% for important North Atlantic regions). Arguably, there is a large overlap of predictors between some of the regions; however, none of the regions feature the exact same predictors. Some regions, for example, the Mediterranean box, could perhaps be subdivided in a western and eastern part, as the grid box level regression suggested that sea ice for example only impacts the eastern part of this box. In that way the predictors could be determined even more precisely.

The added value of the presented article is given by revealing a more comprehensive insight into the physical drivers for serial clustering of windstorms over the European continent on a timescale of more than 100 years. The work presented could proof to be beneficial for the insurance sector as results suggest that fairly simple Poisson GLMs are already able to skilfully estimate whether or not a seasons has the potential of spawning an increased or decreased amount of winter windstorms. Thus, by using large‐scale indices calculated from, fore example, seasonal forecast data, a potential degree of clustering and the number of expected windstorms could be directly obtained from such a model for the subsequent winter season. Additionally, the map of drivers that was presented in section 4.2 provides a useful overview over the spatial structure of prominent large‐scale drivers for the European domain.

## Appendix

**Table A1:** Overview of the potential large‐scale drivers considered for the development of the statistical model of winter windstorms over Europe

Index name	Index source	Additional information
QBO 30 hPa	Observations	Brönnimann *et al*. (2016)
QBO 70 hPa	Observations	Brönnimann *et al*. (2016)
AMO	ERA‐20C (SSTs)	SSTs over North Atlantic (85 –5 W 10°–70 N for DJF season according to http://www.esrl.noaa.gov/psd/data/timeseries/AMO
HSI (horse shoe index)	ERA‐20C (SST)	Index calculation (DJF) according to Renggli (2011)
SSTS	ERA‐20C (SST)	Southern box of HSI
Tdif.Nam	ERA‐20C (SST)	Index calculation according to Wild *et al*. (2015)
W.Atl T	ERA‐20C (SST)	West Atlantic box from Tdif.Nam (DJF)
NINO3.4	ERA‐20C (SST)	Region: 170°–120 W, 5°S–5°N (DJF)
http://nao.is.li	ERA‐20C (MSLP)	Season (DJF) station‐based NAO index
PDO	ERA‐20C (SST)	1st EOF of the DJF Pacific SSTs anomalies (120°E–105 W, 20°–65 N)
West Pacific	ERA‐20C (GPH 700 hPa)	3rd rotated EOF (rEOF) from 700 hPa GPH for Northern Hemisphere. Calculation following (http://www.cpc.ncep.noaa.gov/datateledoc/telecontents.shtml)
PNA	ERA‐20C (GPH 700 hPa)	2nd rEOF from 700 hPa GPH for Northern Hemisphere. Calculation following (http://www.cpc.ncep.noaa.gov/datateledoc/telecontents.shtml)
West.Pacific.10th EOF	ERA‐20C (GPH 700 hPa)	10th rEOF from 700 hPa GPH for Northern Hemisphere. Calculation following (http://www.cpc.ncep.noaa.gov/datateledoc/telecontents.shtml)
EA.WR	ERA‐20C (GPH 700 hPa)	4th rEOF from 700 hPa GPH for Northern Hemisphere. Calculation following (http://www.cpc.ncep.noaa.gov/datateledoc/telecontents.shtml)
EA	ERA‐20C (GPH 700 hPa)	5th rEOF from 700 hPa GPH for Northern Hemisphere. Calculation following (http://www.cpc.ncep.noaa.gov/datateledoc/telecontents.shtml)
SCA	ERA‐20C (GPH 700 hPa)	6th rEOF from 700 hPa GPH for Northern Hemisphere. Calculation following (http://www.cpc.ncep.noaa.gov/datateledoc/telecontents.shtml)
TNH	ERA‐20C (GPH 700 hPa)	7th rEOF from 700 hPa GPH for Northern Hemisphere. Calculation following (http://www.cpc.ncep.noaa.gov/datateledoc/telecontents.shtml)
EP.NP	ERA‐20C (GPH 700 hPa)	8th rEOF from 700 hPa GPH for Northern Hemisphere. Calculation following (http://www.cpc.ncep.noaa.gov/datateledoc/telecontents.shtml)
POL	ERA‐20C (GPH 700 hPa)	9th rEOF from 700 hPa GPH for Northern Hemisphere. Calculation following (http://www.cpc.ncep.noaa.gov/datateledoc/telecontents.shtml)
Sea ice	ERA‐20C (sea ice cover)	Sea ice cover 40°–90 N for DJF
